# Electroconvulsive Therapy and Age: Effectiveness, Safety and Tolerability in the Treatment of Major Depression among Patients under and over 65 Years of Age

**DOI:** 10.3390/ph14060582

**Published:** 2021-06-18

**Authors:** Monika Dominiak, Anna Z. Antosik-Wójcińska, Marcin Wojnar, Paweł Mierzejewski

**Affiliations:** 1 Department of Pharmacology, Institute of Psychiatry and Neurology, Sobieskiego 9, 02-957 Warsaw, Poland; mierzeje@ipin.edu.pl; 2 Department of Psychiatry, Medical University of Warsaw, Nowowiejska 27, 00-665 Warsaw, Poland; antosikwojcinska@gmail.com (A.Z.A.-W.); marcin.wojnar@wum.edu.pl (M.W.)

**Keywords:** electroconvulsive therapy, major depression, treatment resistant depression, elderly, cognitive functions, autobiographical memory

## Abstract

Electroconvulsive therapy (ECT) remains the most effective therapy in treatment-resistant depression. However, the safety of ECT has been consistently questioned, particularly among elderly patients. We assessed the efficacy and safety of ECT in patients before and after 65 years old. The study was conducted between 2015 and 2018 and included 91 patients (61 under and 29 over 65 years old) with major depression undergoing ECT. The Hamilton Depression Rating Scale was used to evaluate efficacy. Cognitive functions were assessed using: MMSE, RAVLT, Trail Making Test, Stroop Test and Autobiographical Memory Interview-Short Form. ECT was more effective in older patients as compared to younger (*p* < 0.001). No serious adverse events were observed in either group. Increased blood pressure and arrhythmias were more common in the older compared to the younger group (*p* = 0.044 and *p* = 0.047, respectively), while disturbances of consciousness did not differ between groups (*p* = 0.820). Most of the cognitive functions remained unchanged compared to baseline, whereas the outcomes of MMSE, RAVLT and Stroop tests showed greater improvements in the older compared to the younger group (all *p* < 0.05). The decline in the retrieval consistency of autobiographical memory was more pronounced in the younger group (*p* = 0.024). ECT is a highly effective, safe and well-tolerated method of treating depression regardless of age.

## 1. Introduction

The chronic and recurrent nature of mood disorders, as well as the often coexisting drug resistance, are current major medical challenges. A significant proportion of patients suffering from major depression despite sequential combination or augmentation treatment strategies do not achieve improvement and meet the criteria of treatment-resistant depression (TRD). Only 60–70% of patients with depressive disorders respond to standard antidepressant treatment (first and second line of treatment) [1,2]. In particular, regarding late-life depression, there is a paucity of data on which evidence-based treatment decisions can be made [3]. This problem grows dynamically with the ageing of the society.

Various strategies to manage the TRD have been evaluated, such as optimizing the dose of the current antidepressant, combining antidepressants, switching to a different antidepressant or augmenting with other medications (including ketamine and esketamine). Each of these options have their own set of potential benefits and drawbacks [4]. As regards the recently introduced esketamine, its applicability in the elderly population, among whom cardiovascular disease often co-occurs, is limited. More clinical and experimental data are also needed with regard to the efficacy, tolerance and security of long-term administration of esketamine.

It is worth emphasizing that not only the occurrence of drug resistance, but also situations where pharmacotherapy causes side effects unacceptable to the patient in the long-term, lead to a shift to treatment methods other than pharmacotherapy. Especially in the population of elderly patients, who often suffer from comorbidities, the problem may be the risk of drug interactions, especially with other drugs taken due to chronic somatic diseases.

This situation necessitates a search for non-pharmacological methods of treatment. Among non-pharmacological therapies, electroconvulsive therapy (ECT) is undoubtedly the most effective treatment for depression [5,6,7,8,9,10]. Transcranial magnetic stimulation (TMS) has few cognitive or somatic side effects but is not as effective in the treatment of psychotic depression or treatment-resistant depression in elderly patients [11,12,13]. Another therapeutic option—vagus nerve stimulation (VNS)—has limited data in older patients but has been shown to be effective in chronic, treatment-resistant adults [14,15]. According to some authors, deep brain simulation (DBS) has also shown promise in geriatric TRD [11]. However, none of the above-mentioned methods is as effective as ECT. Nevertheless, in recent years, there has been also a renewed interest in the chemical induction of seizures through flurothyl and pentylenetetrazol, as they might have less adverse effects on memory than electrically induced seizures [16]. It is also worth noting that the currently available literature does not yet provide convincing evidence to consider ketamine as an equally effective treatment alternative to ECT in patients with TRD [17]. This has prompted many researchers to revisit the oldest non-pharmacological treatment for depression, ECT, but in a new, 21st century setting [18].

It is estimated that the effectiveness of ECT in the treatment of depression may be as high as 80–90% in the case of TRD and remission can be achieved even in 40–50% of patients [19,20,21,22]. Nonetheless, it is not the efficacy of ECT but its safety that has been consistently called into question, particularly in the elderly population [23]. It has been widely recognized that the risk of adverse events is higher in older rather than younger patients undergoing ECT [24]. Studies comparing the safety of ECT in older and younger patients have reported more cardiovascular complications (especially transient increases in blood pressure), as well as cognitive impairment, in older patients [24,25,26]. However, it has been emphasized that it is not age per se but the presence of conditions typical of old age that is the most likely risk factor [27]. As age increases, so does the proportion of comorbidities, including ischemic heart disease, hypertension or cardiac arrhythmias, increasing the overall risk of cardiovascular complications [24]. Given the high efficacy of this treatment for depression, a thorough investigation of the safety and tolerability of this therapy, especially in older patients, seems warranted [28].

The purpose of this study was to compare the efficacy, safety and tolerability of ECT in two age groups of patients treated for depression—younger (under 65 years old) and older (65 years or older). We hypothesized that ECT is a highly effective, safe and well-tolerated method of treating depression regardless of age.

## 2. Results

### 2.1. Study Group

The study enrolled 62 patients under 65 years old (younger group) and 29 patients 65 years old or above (older group) with major depression treated with ECT. Ninety-six percent of the study sample (n = 88) met the criteria of treatment-resistant depression (TRD). TRD was evaluated using the Maudsley Staging Model [29]. This model takes into account symptoms’ severity, the duration of an episode, the number of prior attempts, the usage of the augmentation strategy and previous ECT use. According to this tool, our study sample showed a moderate prevalence of TRD (mean 7.9± 1.4). There were no significant differences between older and younger groups regarding drug resistance. Table 1 presents the clinical and demographic characteristics of the study groups.

The mean age in the younger group was 46.2, and in the older group, it was 70.9 years. The proportions of patients in both age groups did not differ significantly in terms of gender, type of treatment (unilateral/bilateral), drug resistance, presence of psychotic symptoms and the mean number of ECT treatments (all *p*-values > 0.05). However, the groups differed significantly with respect to the presence of somatic diseases, which, as can be expected, were significantly more common in older patients—at least one somatic disease occurred in 65.1% of older patients.

### 2.2. Efficacy of Electroconvulsive Therapy in Relation to Age

The efficacy of ECT was studied in two age groups by comparing the mean change in the HDRS score before and after ECT treatment. The baseline HDRS score (before ECT treatment) differed significantly among the study groups—it was higher in the older group). ANOVA analysis showed a significantly greater reduction in HDRS score after ECT in the older group (group x time: F(1,89) = 14.5; *p* < 0.001; partial η2 = 0.14). There were also significant differences in response rates between compared groups—69% and 89% in the younger and in the older group, respectively (chi2 = 4.44; *p* = 0.035; phi = 0.2). On the other hand, the groups did not differ in terms of remission rates—35.5% and 50.7% (chi2 = 2.15; *p* = 0.141) in the younger and older group, respectively

The efficacy of ECT therapy was also evaluated using secondary scales—BDI and CGI. Significant differences were found between groups in the mean change in the BDI scale (group x time: F(1, 89) = 14.3; *p* < 0.001; partial η 2 = 0.14), as well as in the CGI scale (group x time: F(1, 89) = 16.8; *p* < 0.001; partial η 2 = 0.16).

The mean energy doses used in treatments in patients under and over 65 years of age were also analyzed as a potential confounding variable. The mean energy dose in the older group was significantly higher on average by 134.26 mC (34%) than in the younger age group (*p* = 0.003; 95% CI 223.8-44.68). As regards the mean number of ECT treatments, there were no significant differences between compared groups (*p* = 0.171). The following outcomes are presented in Table 2.

In order to identify predictors of treatment efficacy (as measured by the HDRS scale), a stepwise linear regression analysis was performed. The following variables were included in the model: type of ECT (unilateral/bilateral), patient’s age, gender, drug resistance, baseline severity of depression (baseline score on HDRS), mean energy used in ECT treatments, number of ECT treatments and scores of all applied neurocognitive tests (both before and after ECT treatment).The constructed model explained 13.4% of the variation (R^2^ = 0.134), with the only one explanatory variable—age (β = 0.366)—that contributed significantly to the variation in the final HDRS score. Other variables, including gender and mean energy dose, did not affect the results.

### 2.3. Safety of Electroconvulsive Therapy in Relation to Age

Transient increases in blood pressure and cardiac arrhythmias during the ECT procedures were significantly more frequent in the older group (chi2 = 4.04, *p* = 0.044, phi = 0.25; chi2 = 3.9, *p* = 0.047, phi = 0.22, respectively). There were no significant differences between groups in the percentages of patients experiencing disturbances of consciousness (*p* = 0.820) (Table 3).

### 2.4. Tolerability of Electroconvulsive Therapy in Relation to Age

**Figure 1:**
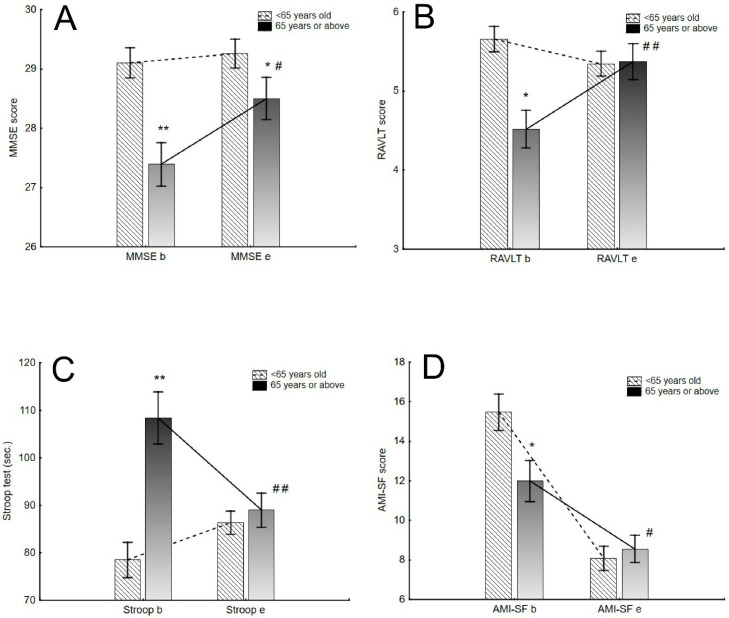
Cognitive assessment in younger (<65 years old) and older (65 years and above) ECT groups at baseline and endpoint. (**A**) MMSE—Mini Mental State Examination; (**B**) RAVLT—Rey Auditory Verbal Learning Test; (**C**). Stroop. (**D**) AMI-SF—Autobiographical Memory Interview Short, b—baseline assessment, e—assessment at endpoint. Vertical bars represent Standard Error. * *p* < 0.05, significant differences between age groups; ** *p* < 0.01, significant differences between age groups; # *p* < 0.05, significant differences after ECT treatment between age groups; ## *p* < 0.01, significant differences after ECT treatment between age groups.

Twenty-seven patients over and 58 under 65 years old were assessed in terms of neurocognitive functioning. At baseline, the over 65 group had lower scores on MMSE, RAVLT, Stroop test, TMT, verbal fluency and AMI-SF (specific memory subtest regarding specific events in the last year) (all *p* < 0.05). Electroconvulsive therapy was found to improve to a greater extent the results of the following tests in the group of older patients as compared to the younger group: MMSE (ATS(1) = 12.1; *p* = 0.022, p_corr_ = 0.04, partial η2 = 0.07); RAVLT-subtest A1 (F(1,81) = 16.1 *p* < 0.001, p_corr_ = 0.0002, partial η2 = 0.16) and Stroop test (F(1,81) = 17.1, *p* < 0.001, p_corr_ = 0.007, partial η2 = 0.17). In terms of autobiographical memory, the results of the AMI-SF test showed a significantly lower decline in retrieval consistency in this memory type in older patients compared to younger ones (F(1,37) = 7.1, *p* = 0.011, p_corr_ = 0.024, partial η2 = 0.15) (Figure 1). There were no significant differences between groups in other tests (all values *p* > 0.05). Additional ANOVA analysis considering also the type of ECT (bilateral/unilateral) revealed no significant influence of this variable on the neurocognitive tests (all *p* > 0.05), except for the RAVLT test, where the interaction with time and age group in influencing the increase in RAVLT test score was noted (F(1.81) = 7.93, *p* = 0.0061, p_corr_ = 0.01).

## 3. Discussion

This study showed that ECT is an effective and safe treatment for TRD in all ages. An important conclusion that might be also drawn from this study is that the efficacy and tolerability of ECT was even better in the group of greatest concern—older depressed patients (over 65 years of age). This result is partially consistent with data in the literature. The efficacy of ECT in the older patients was higher than in the younger age groups [26,30,31,32,33,34,35]. Recent reviews and meta-analyses also seem to support these findings [27,28,36]. Nevertheless, not all researchers agree on this. For instance, a study by Socci et al. [37] showed similar efficacy of ECT in older and younger patients. Similarly, retrospective analyses [25,38] and meta-analyses [39] did not find a significant association between the efficacy of ECT and older age. It has been also highlighted that it is not age per se, but rather the different clinical picture of depression in the elderly (more frequent occurrence of suicidal thoughts, longer duration of an episode, presence of psychotic symptoms or very severe psychomotor retardation) that may be factors determining the higher efficacy of ECT in this population [40]. Some authors have also postulated that the higher efficacy of ECT in a population of older depressed patients may be due to the higher prevalence of somatic symptoms and their reflection in scores on the HDRS [41]. In our study, however, we have applied two additional scales (BDI and CGI) assessing the severity of symptoms and obtained results indicating the higher efficacy of ECT in older patients. As the mean energy dose was significantly higher in the older group (due to the higher seizure threshold in elderly patients), we also consider this variable as a potentially confounding factor that might influence the results. The regression analysis showed that the higher efficacy of ECT treatment was only significantly associated with older age, and not with the energy dose or with any other variable analyzed (including prevalence of psychotic symptoms, severity of depression, stage of TRD, type of ECT). Therefore, based on this study, the exact mechanism that would account for the higher efficacy of ECT in elderly compared to younger patients remains unclear. This issue could be a subject for further research. Nonetheless, for clinical reasons, it is extremely important to state that ECT is highly effective in patients with TRD, including a particularly responsive group of older depressed patients. Thus, ECT may be a very good alternative to pharmacotherapy, which is often ineffective or causes frequent side effects.

As regards the safety of ECT, the results of this study have confirmed that it is a very safe method of treatment, irrespective of age. Although transient increases in blood pressure and cardiac arrhythmias were significantly more common in the older group, they were mild and transient and did not require any particular treatment. The cardiovascular side effects noted in this study among older patients are slightly lower than reported in other studies [25,26]. Somatic diseases, including hypertension, ischemic heart disease and cardiac arrhythmias, were also significantly more common in the over-65 population compared to the younger population. The higher prevalence of cardiovascular side effects in this group therefore seems to be a logical consequence of a higher baseline burden of somatic diseases. Nevertheless, it should be stressed that no serious side effects or complications were reported in either the younger or older group. One can also find in the literature reports concerning a particularly frequent complication of ECT especially seen in the elderly population—disturbances of consciousness [42]. On the contrary, Antosik-Wójcińska and Swiecicki [24] report that disturbances of consciousness occur in this population with a similar frequency as in younger patients. The results of this study are in line with the latter work as disturbances of consciousness were not significantly more frequent in older patients. Similarly, memory impairment appears to be a very common complication of ECT, especially among the elderly [24,25,43]. At this point, it is worth noting that cognitive impairment is prevalent at baseline in older patients—as one of the depressive symptoms, mild cognitive impairment or dementia. The neurocognitive assessment performed in this study confirmed the good tolerability of ECT, including among a group of older depressed patients, often perceived as particularly vulnerable. Most of the cognitive functions remain unchanged compared to baseline assessment, whereas general cognitive performance, verbal auditory memory, working memory and executive functions improved significantly more in older patients compared to younger ones. Our results are partly consistent with the data in the literature. For instance, Meyer et al. [36] and Obbels et al. [44] reported the good tolerability of ECT among elderly patients, and Socci et al. [37] observed that older age groups showed a significantly greater improvement in MMSE score than younger age groups. Similarly, Verwijk et al. [45] found an improvement in general cognitive performance (assessed by the MMSE test), verbal auditory memory and verbal fluency in older patients after ECT course. Furthermore, improvements in cognitive function have also been observed in depressed patients with baseline cognitive impairment [46,47]. In our study, patients over 65 years of age showed also significantly less decline in the retrieval consistency of autobiographical memory (concerning specific events in the past year) than younger patients, although it was noted in both groups. The problem with the decline in this particular memory and its assessment is being widely discussed in the literature [48,49,50].

It is worth noting that in the present study, as in all other similar studies, the elderly patient group obtained lower scores on most neurocognitive tests at baseline compared to younger patients. Given both the issue of age and the differences in the clinical picture of depression in old age (frequent memory impairment), this fact might be surprising. Nevertheless, the results of the study indicate that some cognitive functions improve after ECT. The fact that this applies notably to the elderly is of particular clinical significance.

An entirely different matter is also the fact that unilateral ECT is suggested to have better cognitive outcomes [20,50] and was shown to be associated with greater improvement in some cognitive functions [48]. In our study, the compared age groups did not differ in terms of the proportion of patients receiving a given type of ECT; thus, this variable did not affect the results. Nevertheless, additional analyses revealed a greater improvement in verbal fluency (as measured by the RAVLT test) in patients treated with unilateral ECT.

When choosing a therapeutic option in the case of an inadequate response to an antidepressant, clinicians should take each patient’s predispositions and individual needs into consideration. As pharmacotherapy in many cases turns out to be insufficient, other strategies involving non-pharmacological methods, including ECT, should always be considered. Since the present trend in neuromodulation therapies is to apply a personalized treatment [51], current challenges include searches of predictive factors allowing for the determination of the most beneficial therapy for a given patient. Based on the results of this study, it can be concluded that age is a very important predictive factor when applying ECT. It implies the greater efficacy of this therapy, very good tolerability and safety. On the other hand, another, somewhat indirect, finding based on the size of the study sample is that ECT is used relatively rarely in older depressed patients.

### Limitations

The compilation of the entire sample in terms of age reflects the inflow of depressed patients consecutively eligible for ECT treatment in a given time period. Therefore, the studied groups were not equal. Nevertheless, the age groups did not differ significantly in any of clinical parameters (with the exception of a higher percentage of somatic diseases in the older group). We also used various statistical methods to account for this in the analysis. As positive results were obtained in most analyzed parameters, the study samples appeared to be sufficient to capture significant differences between groups. Nonetheless, the results should be interpreted in light of the above limitation.

## 4. Materials and Methods

### 4.1. Study Design

This prospective study was conducted between the years 2015 and 2018 at the Institute of Psychiatry and Neurology (IPiN), Warsaw, Poland. Informed consent was obtained from all participants involved in the study. The study was conducted in accordance with the Declaration of Helsinki, and the protocol was approved by the Bioethics Committee at the Institute of Psychiatry and Neurology in Warsaw (resolution No. 23/2015). The inclusion criteria were: patients over 18 years of age, hospitalized with a diagnosis of major depression (according to ICD-10 classification) and qualified for ECT treatment, who had given their informed written consent for the participation in the study. Patients were excluded if they had been treated using ECT in the past 6 months (to exclude the possible long-term impact of the ECT on cognitive functions) or were unable to give written informed consent to participate in the study. The final study sample comprised patients who met inclusion criteria and did not meet exclusion criteria and reflected the population of depressed patients that had received ECT treatment in IPiN during this period of time. The study population consisted only of hospitalized patients as, according to Polish law regulations, ECT procedures can be performed only in inpatient settings. The total sample was further split into two groups with regard to the participants’ ages. In the present study, a cut-off point of 65 years was used as, in most previous works on this topic, such an age limit was assumed [52].

### 4.2. Clinical Assessment

The clinical assessments were performed by clinical psychiatrist with experience in affective disorders. The diagnoses were established according to the ICD-10 criteria by consensus between two psychiatrists. Mental and somatic states were evaluated prior to ECT and 2 weeks after the end of the ECT course.

The methodology of clinical assessment was as previously described in the work of Dominiak et al. [30,48]. To assess the efficacy of ECT and compare it between groups, we used the Hamilton Depression Rating Scale (HDRS-21) as the primary measure. The primary outcome was the mean change in the HDRS score and the secondary outcomes were the response and remission rates. Response to treatment was defined as a reduction in HDRS score by at least 50% from baseline, and remission as 8 or less on the HDRS scale. Additionally, the Beck Depression Inventory (BDI) and Clinical Global Impression Scale (CGI) were also applied as secondary efficacy measures.

The somatic state was monitored throughout the entire course of the treatment and included both the assessment during the ECT procedure and up to 5 h after (BP, ECG, pulsoxymetry, consciousness) and daily observations during the entire ECT course (BP, consciousness, ECG in case of relevant complaints). ECT treatment was discontinued in the event of serious adverse effects or withdrawal of the patient’s consent. The neuropsychological assessment was performed by a clinical neuropsychologist with experience in affective disorders and in geriatric psychiatry. The following tools were used in order to assess cognitive functions: Mini Mental State Examination (MMSE), Rey Auditory Verbal Learning Test (RAVLT), Trail Making Test, Rey-Osterrieth Complex Figure, Stroop tests, Verbal Fluency Test, Autobiographical Memory Interview-Short Form (AMI-SF) [53].

### 4.3. Procedures

Before the ECT course, all psychotropic medications (benzodiazepines, anticonvulsant medications, lithium, antidepressants and antipsychotics) were discontinued. Patients continued with medications taken for somatic conditions (including blood pressure-lowering, cardiovascular, lipid-regulating, glaucoma, thyroid and prostate hypertrophy medications). Participants received a brief pulse formula-based ECT, unilateral or bilateral treatments. ECT treatments were performed with the Somatics Thymatron-System-IV apparatus, using a current intensity of 0.9 A and a pulse width of 0.5 ms. The amount of the electric charge administered to the patient during the first ECT treatment was determined based on the patient’s age. Procedures took place twice a week until a patient reached remission (≤8 points in HAMD-21 scale) or plateau (no further improvement in four consecutive sessions), with a maximum of 16 sessions. The ECT treatments were performed under general anesthesia, with oxygenation with 100% oxygen for 1 min, atropine (0.5–1 mg), propofol (2 mg/kg) or thiopental (3–4 mg/kg). There was no difference in the use of these two anesthetics between groups (*p* = 0.43). For muscle relaxation, suxamethonium chloride (0.25–1 mg/kg) was given.

### 4.4. Data Analysis

Analyzed groups were compared using analysis of variance (ANOVA) with repeated measurement, ANOVA-type statistic (ATS), Student t-test, Mann–Whitney U test and Chi-square test with Yates correction. The correction for multiple comparisons of Benjamini–Hochberg (p_corr_) was applied [54]. Missing data (concerning some of the neurocognitive tests) were handled using the multiple imputation method. Descriptive statistics were calculated using averages and standard deviations or the median and interquartile range. Data distribution was analyzed using Shapiro–Wilk test and skewness analysis. Relationships between variables were verified using regression and correlation methods. The effect of age, gender, diagnosis, duration of illness, presence of psychotic symptoms, ECT electrode placement, mean charge (mC) and somatic comorbidities on the efficacy of ECT was also analyzed. Statistical significance was set at *p*-value < 0.05. The analyses were performed using IBM SPSS Statistics 23, TIBCO Statistica 13.3 and R 3.5.2.

## Figures and Tables

**Table 1:** Clinical and demographic characteristics of participants.

Parameter	Under 65 Years Old	65 Years or Older	*p*
Number of patients n (%)	62 (68,1%)	29 (31,9%)	
Mean age (in years) (SD)	46.2 (12.4)	70.9 (5.07)	<0.001
Gender F/M	F–33 M-29	F–14 M-15	0.659
Type of ECT unilateral/bilateral	31/31	15/14	0.878
Score according to MSM Mean (SD)	9.2 (1.5)	8.9 (1.2)	0.354
Drug resistance—number of patients (n)	60	28	0.324
Number of patients with psychotic symptoms n (%)	16 (25%)	9 (31,0%)	0.602
Number of patients with at least one somatic disorder n (%)	17 (27%)	19 (65%)	0.0005
HDRS b M (SD)	28.3 (6.43)	30.1 (5.81)	0.082
HDRS e M (SD)	11.5 (4.50)	8.2 (3.96)	0.001

n—number, (%)—percent, M—mean, SD—standard deviation, p—*p*-value from t-test, Chi-square test with Yates correction, or from U Mann–Whitney test, MSM—scoring of treatment-resistant depression according to Maudsley Staging Model, HDRS b—Hamilton Depression Rating Scale baseline result (before treatment), HDRS e—Hamilton Depression Rating Scale result at the endpoint (after treatment).

**Table 2:** Mean energy charge and number of ECT treatments by age.

	<65 Years Old (*n* = 62)		≥65 Years Old (*n* = 29)				95% *CI*		
	*M*	*SD*	*M*	*SD*	*t*	*p*	*LL*	*UL*	*d* Cohen
Charge (mC)	405.64	194.19	539.9	213.20	2.978	0.003	−223.8	−44.68	0.706
Number of ECT treatments	11.03	2.03	10.31	2.87	1.379	0.171	−0.473	1.916	0.310

**Table 3:** Adverse effects of ECT by age groups.

Adverse Effects	<65 Years Old (*n* = 62)	≥65 Years Old (*n* = 29)	*p*-Value
Blood pressure elevation	11.3% (*n* = 7)	24.1 % (*n* = 9)	0.044
Cardiac arrythmias	4.8% (*n* = 3)	20.7 % (*n* = 6)	0.047
Disturbance of consciousness	6.4% (*n* = 4)	10.3% (*n* = 3)	0.820
Headaches	11.3% (*n* = 7)	10.3% (*n* = 3)	0.821

*p*-values from the Chi-square test with Yates correction.

## Data Availability

The data presented in this study are available on request from the corresponding author. The data are not publicly available due to privacy or ethical concerns.
